# Genome-wide identification and analysis of long noncoding RNAs (lncRNAs) during seed development in peanut (*Arachis hypogaea* L.)

**DOI:** 10.1186/s12870-020-02405-4

**Published:** 2020-05-06

**Authors:** Xingli Ma, Xingguo Zhang, Sy Mamadou Traore, Zeyu Xin, Longlong Ning, Ke Li, Kunkun Zhao, Zhongfeng Li, Guohao He, Dongmei Yin

**Affiliations:** 1 College of Agronomy, Henan Agricultural University, Zhengzhou, 450002 China; 2 College of Agriculture, Environment and Nutrition Sciences, Tuskegee University, Tuskegee, 36088 AL USA

**Keywords:** Peanut, lncRNA, Seed development, RNA-Seq, Transcriptional regulation

## Abstract

**Background:**

Long noncoding RNAs (lncRNAs) have several known functions involving various biological regulatory processes in plant. However, the possible roles of lncRNAs during peanut seed development have not been fully explored.

**Results:**

In this study, two peanut recombinant inbred lines (RIL_8_) that differ in seed size were used to investigate comprehensive lncRNA profiles derived from the seed development at 15 and 35 days after flowering (DAF). We identified a total of 9388 known and 4037 novel lncRNAs, from which 1437 were differentially expressed lncRNAs. Interestingly, the expression patterns of a number of lncRNAs can be very different between two closely related inbred lines and these lncRNAs were expressed predominantly in only one RIL at 35 DAF. Some differentially expressed lncRNAs were found related to putative cis-acting target genes and predicted to be involved in transcription, transport, cell division, and plant hormone biosynthesis. The expression patterns of several representative lncRNAs and 12 protein-coding genes were validated by qPCR. Same expression pattern was observed between most lncRNAs and their target genes. 11 lncRNAs, XR_001593099.1, MSTRG.18462.1, MSTRG.34915.1, MSTRG.41848.1, MSTRG.22884.1, MSTRG.12404.1, MSTRG.26719.1, MSTRG.35761.1, MSTRG.20033.1, MSTRG.13500.1, and MSTRG.9304.1 and their cis-acting target genes may play key roles in peanut seed development.

**Conclusions:**

These results provided new information on lncRNA-mediated regulatory roles in peanut seed development, contributing to the comprehensive understanding of the molecular mechanisms involved in peanut seed development.

## Background

In recent years, the use of the next generation sequencing approaches revealed that transcription in eukaryotes is complex. A large number of eukaryotic genomes are universally transcribed producing coding and non-coding RNAs (ncRNAs). Non-coding RNAs (ncRNAs) are a class of RNAs that cannot code for proteins; however, they play important regulatory roles in numerous biological processes [1]. ncRNAs can be classified based on their lengths into small RNAs (< 200 nt) and long non-coding RNAs (lncRNAs, longer than 200 nt). Small RNAs are further classified as microRNAs (miRNAs), small nucleolar RNAs (snoRNAs), small interfering RNAs (siRNAs), and small nuclear RNA (snRNAs) [2]. LncRNAs are categorized as long intergenic noncoding RNAs (lincRNAs), natural antisense transcripts (NATs) and intronic RNAs (incRNAs) according to the genomic location and context [3, 4].

LncRNAs exhibit tissue and cell-specific expression patterns and tend to show poor conservation across different species [5, 6]. Previous studies found that lncRNAs involve in several activities, including gathering, transporting proteins, regulating promoter activities through proximal *cis*-acting or *trans*-acting sequences, and epigenetic modification, silencing or repression [7, 8]. LncRNAs also involve in growth and development, disease occurrence in mammals [9, 10].

Several biological functions of lncRNAs have been characterized in various plants. For example, *COOLAIR* and *COLDAIR* regulate the expression of *FLOWERING LOCUS C* (*FLC*) which determines *Arabidopsis* flowering time [11, 12]. In hybrid rice, photoperiod-sensitive male sterility is correlated with a lncRNA, known as long-day–specific male-fertility–associated RNA (LDMAR) [13]. In cotton seedlings, two lncRNAs, GhlncNAT-ANX2 and GhlncNAT-RLP7, provided an enhanced resistance against fungal pathogens *Verticillium dahliae* and *Botrytis cinereal* [14]. In *Medicago truncatula*, the lncRNA *Enod40* interacts with soil rhizobia for nodule formation by inducing a re-localization of a nuclear RBP [15]. Despite this progress, there is still a paucity of studies addressing the role of the lncRNA in plants, more importantly peanut. To date, genome-wide analyses of lncRNAs have been carried out in several plants, but the numbers and characteristics of lncRNAs involved in seed development have not yet been explored in peanut.

Peanut (*Arachis hypogaea* L.), an allotetraploid species (2n = 4x = 40; AABB), is an important crop grown worldwide for both oil and protein production. Peanut seed size is a main agronomic trait for breeders, therefore understanding the molecular mechanisms underlying the development of the peanut seed is a high priority for peanut researchers and breeders. Recent research studies have focused on the identification of some important genes contributing highly to peanut seed development [16]. Moreover, the function of several miRNAs and their target genes involved in peanut seed development has been characterized [17]. However, there is no report provided any information on the involvement of lncRNAs and their target genes in peanut seed development mechanisms. In this study, we used two peanut RILs to gain a better understanding of the function of lncRNA in peanut seed development. The expression profiles of lncRNAs at 15 and 35 days after flowering (DAF) were characterized in two sister lines from a RIL_8_, where line 8106 has medium-sized pods and line 8107 has super-large pods with different seed sizes. We identified and validated lncRNAs in peanut; then identified the differentially expressed lncRNAs (DELs) by comparing and analyzing the expression profile of the lncRNAs in different seed developmental stages between the two peanut RILs; and therefore, understanding the possible roles of DELs in peanut seed development.

## Results

### Sequencing of peanut lncRNAs

In order to investigate the dynamic variation of lncRNAs during peanut seed development, the whole-transcriptome strand-specific RNA sequencing for two peanut RILs at two seed developmental stages (15 DAF and 35 DAF) was performed with three biological replicates. In total, more than 100 million raw reads were generated by high-throughput sequencing. Fast QC with a phred-like algorithm provided a mean quality score (Q30%) greater than 95%, indicating the RNA-seq data was highly reliable. From the raw reads, more than 97% reads were clean reads (Additional file 1: Table S1).

### Identification and characterization of lncRNAs in peanut

**Fig. 1:**
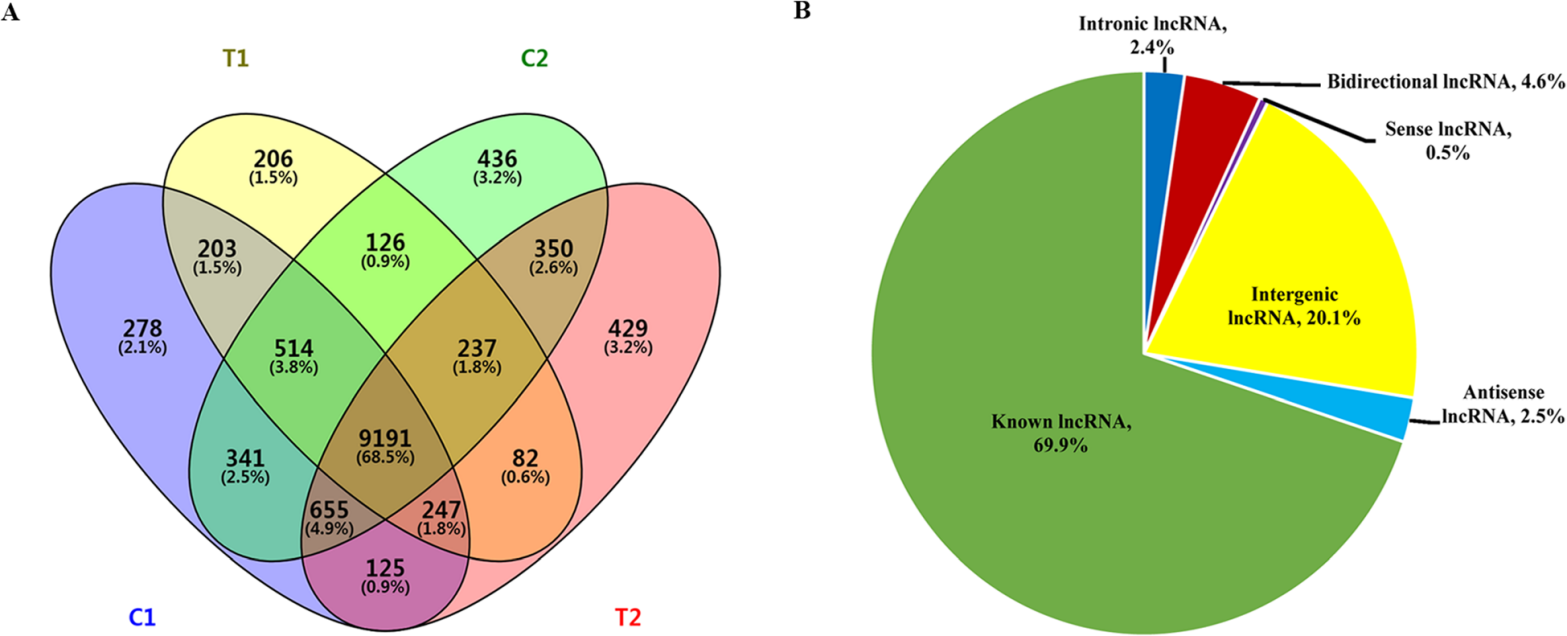
Expressed lncRNAs in two peanut RILs. Venn diagrams showing the number of common and specific lncRNAs in the four libraries (**a**). Pie chart showing composition of different types of lncRNAs (**b**)

The clean reads were mapped to the two diploid Arachis reference genomes (*A. duranensis* and *A. ipaensis*) using the TopHat. Then, the transcripts were assembled and annotated using the StringTie. Mostly, 73,180, 72,725, 74,702, 74,324, 74,027, 73,964, 69,645, 69,155, 70,184, 70,516, 69,935, and 72,215 unique mRNAs from the twelve cDNA libraries were identified, including C1(RIL8106-15DAF), C2(RIL8107-15DAF), T1(RIL8106-35DAF), and T2 (RIL8107-35DAF), each with three replicated samples, respectively (Additional file 2: Table S2). The remaining transcripts were filtered according to their length and coding potential; transcripts shorter than 200 bp were discarded, and transcripts with potential CPC score > − 1 and CNCI score > 0 were removed [18, 19]. The remaining transcripts were considered as lncRNAs, from which, 9528, 9429, 10,177, 10,274, 10,089, 10,131, 9005, 8867, 9044, 9388, 9398, and 9693 unique lncRNAs were identified from the twelve samples, respectively (Additional file 2: Table S2). In total, 13,425 unique lncRNAs were identified in this study (Additional file 3: Table S3). The numbers of lncRNAs found in the two RILs at 15 DAF (11,554 in C1 and 11,850 in C2) were considerably higher than those detected at 35 DAF (10,806 in T1 and 11,316 in T2), and 9191 lncRNAs were common to all four samples (Fig. 1a).

**Fig. 2:**
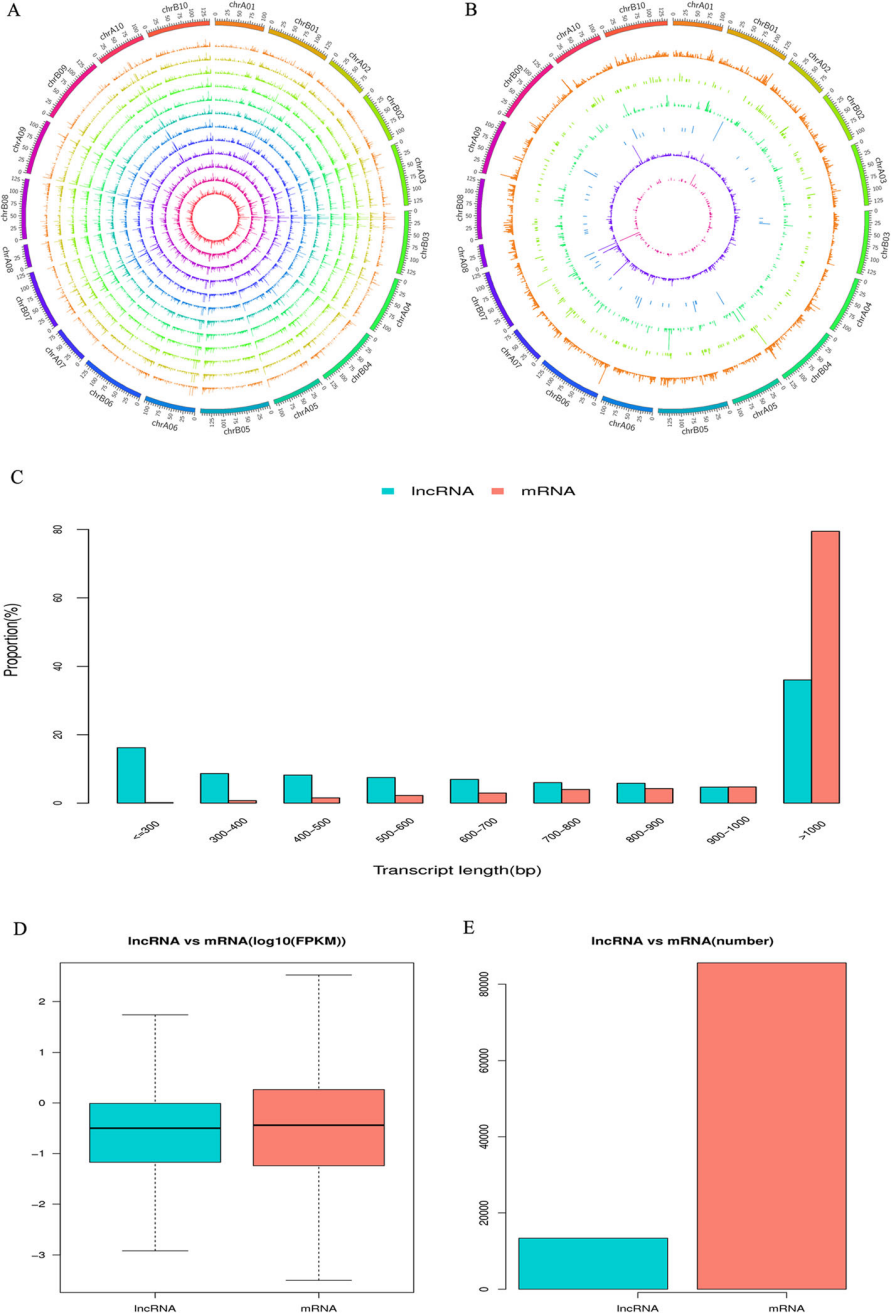
The expression level of lncRNAs along the twenty peanut chromosomes (**a**); comprising twelve concentric rings, and each corresponding to a different sample including C1(RIL8106-15DAF), C2(RIL8107-15DAF), T1(RIL8106-35DAF), and T2 (RIL8107-35DAF) from outer to inner, respectively. Distribution of different types of lncRNAs (**b**). The known, intronic, bidirectional, sense, intergenic, and antisense lncRNAs are represented by different concentric rings according to the loci of lncRNAs in the genome. Length distribution of lncRNAs (**c**). Expression level (**d**) and number (**e**) of lncRNAs and mRNAs in samples

All lncRNAs were mapped to the 20 chromosomes of the peanut genome. Our result indicated that the lncRNAs were evenly distributed across these chromosomes without a preference of location (Fig. 2a and b). According to the genomic locations of these lncRNAs, 2693 intergenic (20.1%), 616 bidirectional (4.6%), 340 antisense (2.5%), 320 intronic (2.4%), 68 sense lncRNAs (0.5%) were identified. In addition, 9388 known lncRNAs (69.9%) were also detected in this study (Fig. 1b). The estimation of the length of these lncRNAs revealed that most lncRNA possessed more than 1000 bp (Fig. 2c). Furthermore, the assessment of the expression level of each transcript using fragments per kilobase of exon model per million mapped reads (FPKM) showed that the overall expression level and number of lncRNAs were lower than those of mRNAs (Fig. 2d and e). The result was consistent with previous study in the upland cotton [20].

### Analysis of differentially expressed lncRNAs in peanut

**Fig. 3:**
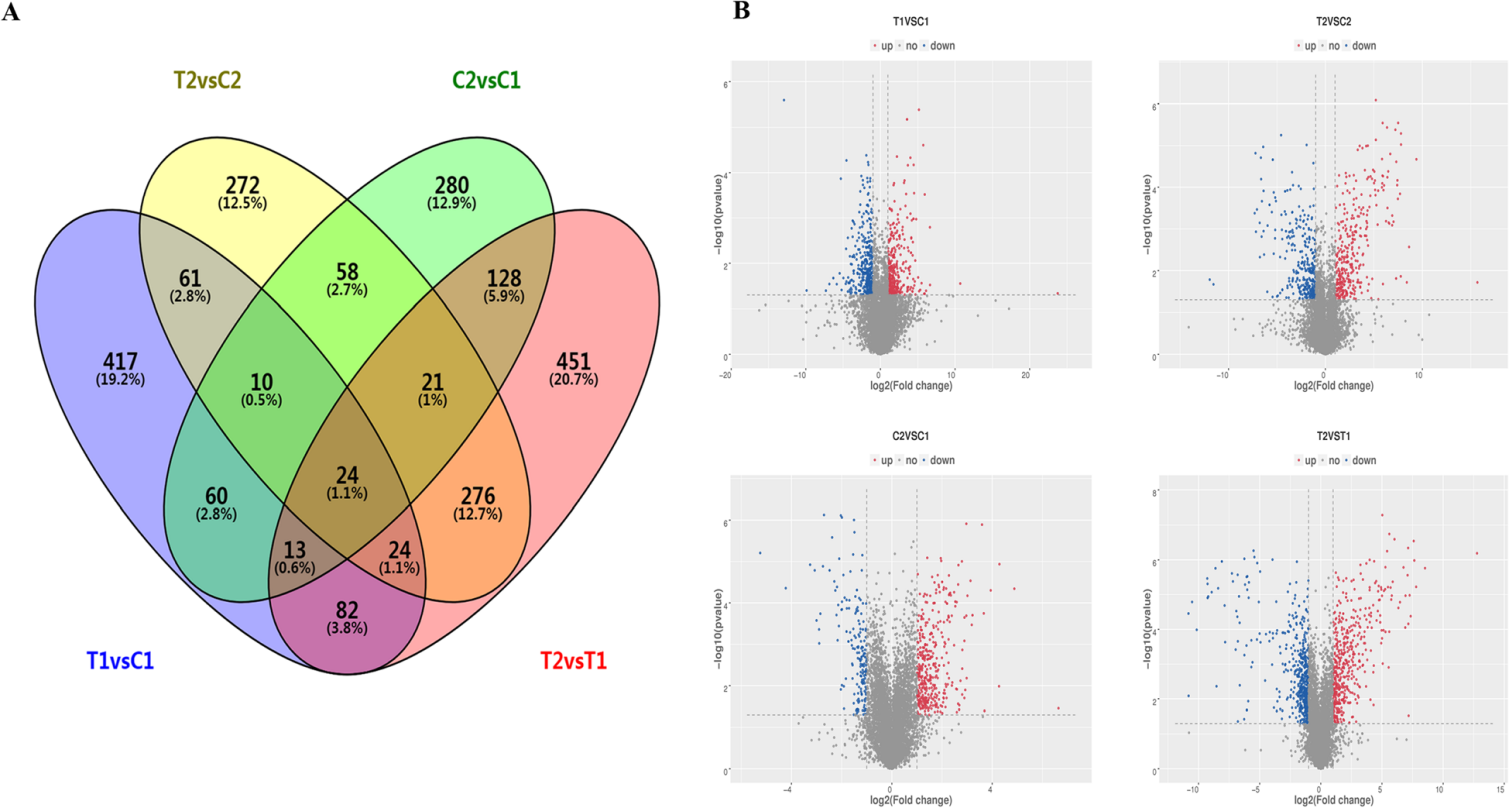
Differentially expressed lncRNAs in two peanut RILs. Venn diagrams showing the number of common and specific lncRNAs in comparisons of the four libraries (**a**). Volcanic diagrams showing the number of differentially-expressed lncRNAs in each comparison (**b**). The red dots indicate lncRNAs with significant up-regulation, and the blue dots indicate lncRNAs with significant down-regulation

**Fig. 4:**
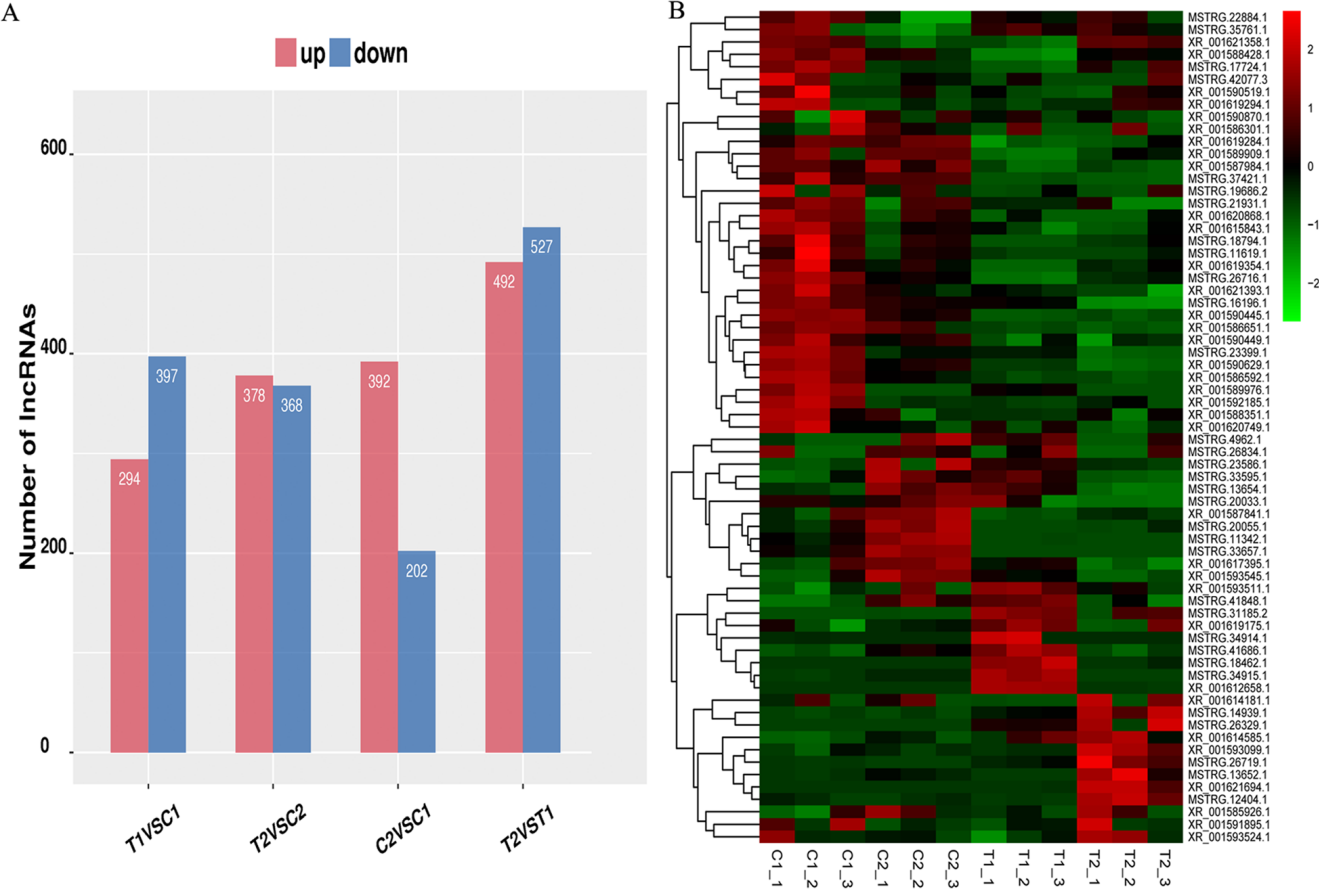
Regulation of differentially expressed lncRNAs in four different comparisons (**a**). Clustering of 67 differentially expressed lncRNAs in the two peanut RILs (**b**)

To analyze the difference of expression of lncRNAs at 35 DAF and 15 DAF between the two peanut RILs, we compared the normalized expression (FPKM) of lncRNAs amongst all libraries. The following criteria were used to identify differentially expressed lncRNAs in the comparison of different groups: (1) log2 (fold change) > 1 or log2 (fold change) < − 1, and (2) statistical significance (*p* value < 0.05). We finally identified 2178 differentially expressed lncRNAs from four different comparisons. Among these, 594, 1019, 691, and 746 differentially expressed lncRNAs were found in comparisons of C2 vs. C1, T2 vs. T1, T1 vs. C1, and T2 vs. C2, respectively, and 24 differentially expressed lncRNAs were common in the four comparisons (Fig. 3 and Additional file 4: Table S4–1, S4–2, S4–3, and S4–4). Moreover, the differentially expressed lncRNAs in four comparisons between two RILs showed that the number of up- and down-regulated lncRNAs was close, such as 294 up and 397 down-regulated lncRNAs in T1 vs. C1, 378 up and 368 down in T2 vs. C2, and 492 up and 527 down in T2 vs. T1 (Fig. 4a). However, significant difference of 392 up- and 202 down-regulated lncRNAs was identified in C2 vs. C1 where the large-seeded line (RIL8107) compared with the medium-seeded line (RIL8106) at the 15 DAF stage (Fig. 4a).

In addition, we clustered the 67 differentially expressed lncRNAs on the basis of their expression patterns in the two developmental stages between the two peanut RILs. Our result showed a difference of expression between the lncRNAs at 35 DAF stage in the two RILs. For example, XR_001590629.1 and MSTRG.37421.1 were down-regulated, while XR_001593099.1 was up-regulated in the two RILs (Fig. 4b). Interestingly, many lncRNAs were expressed predominantly in only one of the RILs at the 35 DAF stage. Fifteen (15) lncRNAs expression, including XR_001593511.1, MSTRG.18462.1, MSTRG.31185.2, MSTRG.34915.1, MSTRG.41848.1, were up-regulated only in medium-size seed line of RIL8106, while only nine (9) lncRNAs including XR_001614585.1, XR_001593524.1, MSTRG.22884.1, XR_001621358.1, XR_001621694.1, MSTRG.12404.1, MSTRG.13652.1, MSTRG.26719.1, and MSTRG.35761.1 were up-regulated only in large-size seed line of RIL 8107 (Fig. 4b). These results suggested that these differentially expressed lncRNAs might play an important role in regulating peanut seed development.

### Validation of differentially expressed lncRNAs

**Fig. 5:**
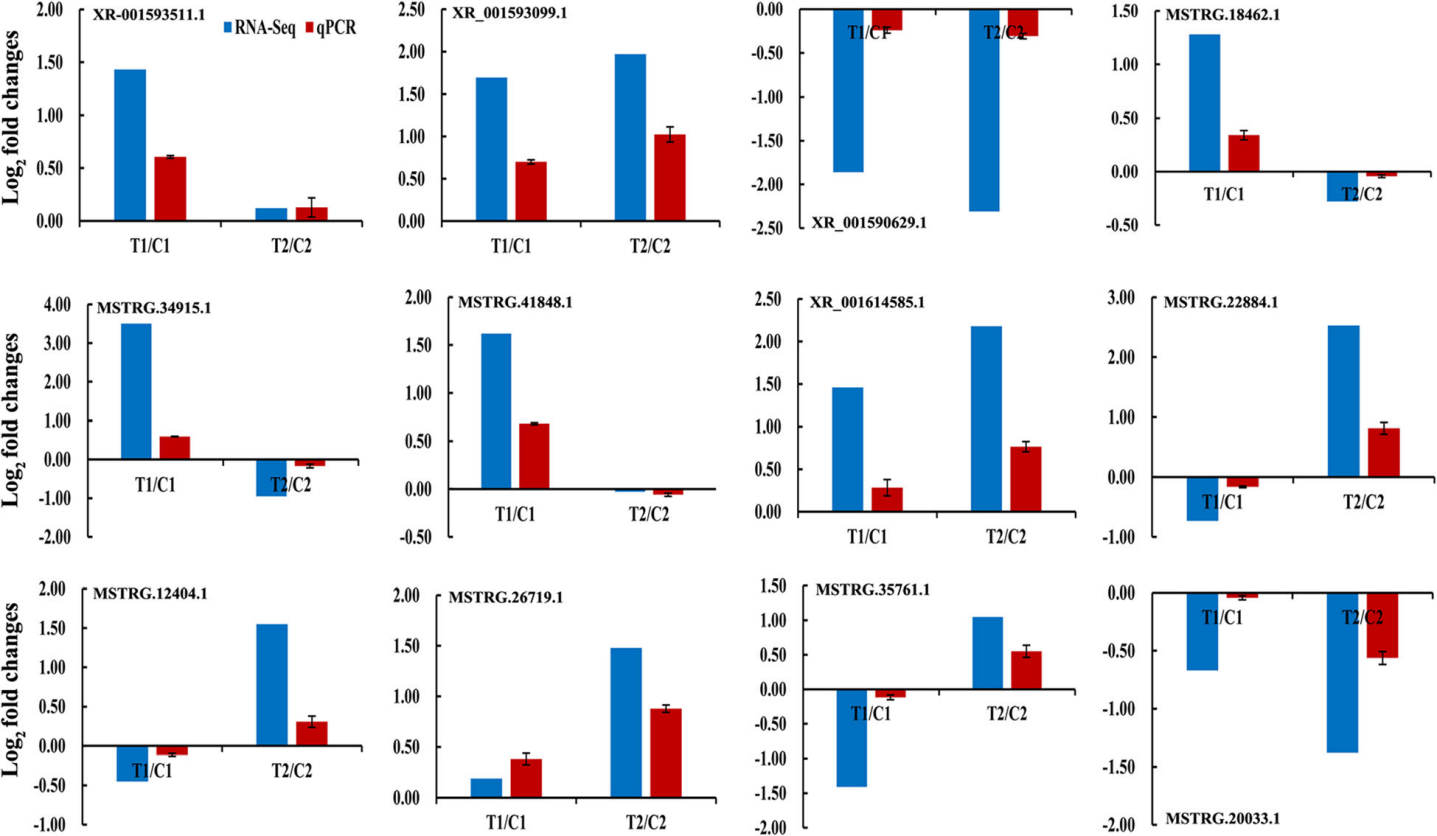
Comparisons of the expression levels of 12 lncRNAs between RNA-seq and qPCR. The expression levels were normalized to the expression of Actin in qPCR. Red indicates the fold-changes of lncRNA expression levels determined by qPCR. Blue indicates the lncRNA expression fold-changes generated from the RNA-seq. The experiments were repeated three times, and vertical bars indicate the standard errors

To confirm the data from the RNA-seq, we randomly selected 12 lncRNAs to verify their expression patterns by qPCR (Fig. 5). The qPCR results of these lncRNAs were consistent with those by high-throughput sequencing. For instance, the expression level of lncRNA XR_001593099.1 was confirmed as up-regulated in both peanut RILs, while lncRNA XR_001590629.1 was down-regulated in both RILs. However, lncRNAs XR-001593511.1, MSTRG.18462.1, MSTRG.34915.1, and MSTRG.41848.1 showed up-regulated expression only in RIL8106 by two methods. Similarly, lncRNAs XR_001614585.1, MSTRG.22884.1, MSTRG.12404.1, MSTRG.26719.1, and MSTRG.35761.1 displayed up-regulated only in RIL8107 by both methods. Moreover, the result from the qPCR method also verified that lncRNA MSTRG.20033.1 was down-regulated predominantly in RIL8107 as showed by RNA-seq. These results indicate that RNA-seq analysis is highly reliable prediction of lncRNA expression patterns.

### Function of differentially expressed lncRNAs during peanut seed development

**Fig. 6:**
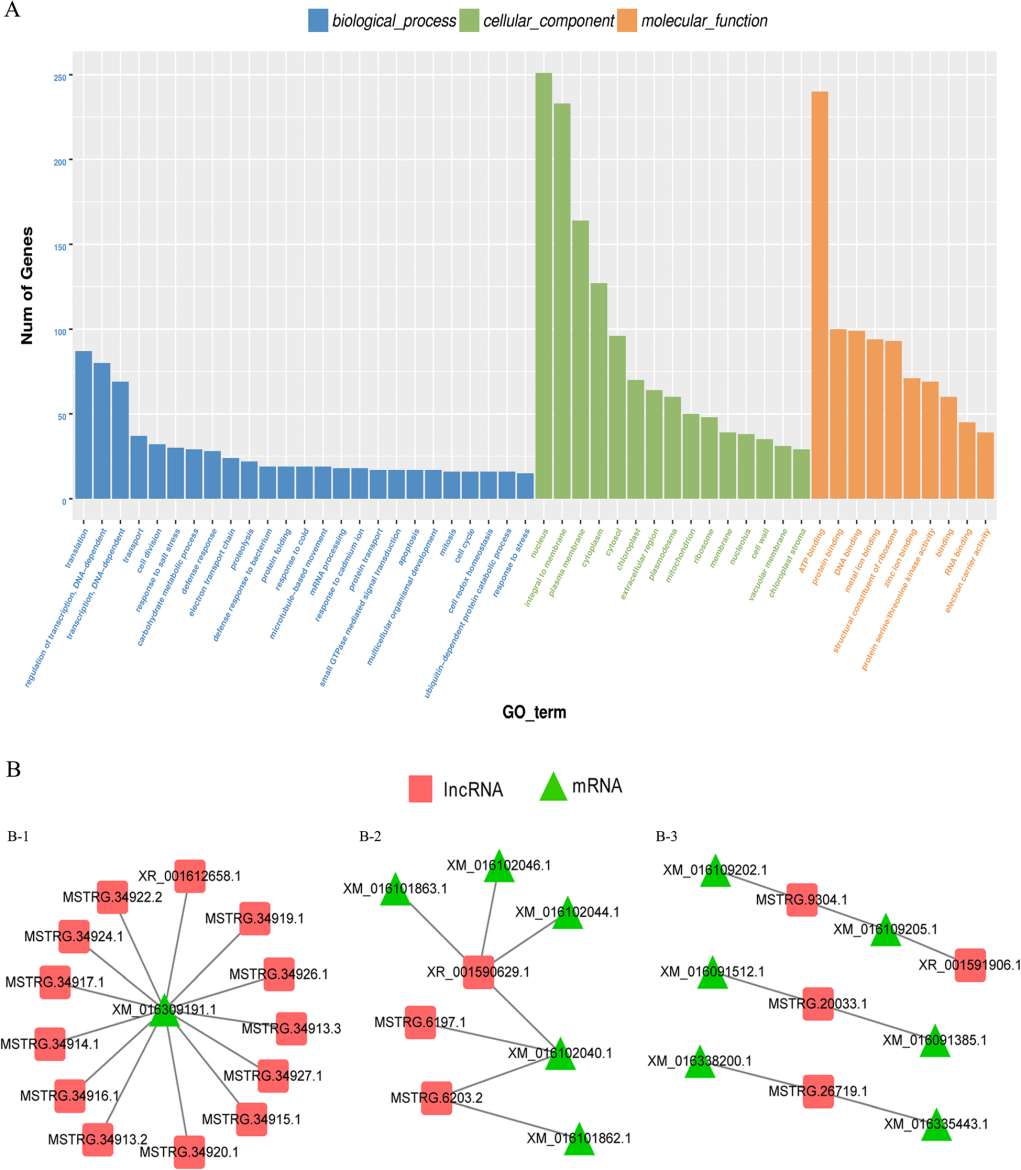
Gene Ontology analysis of co-expressed protein-coding genes with the differentially expressed lncRNAs (**a**). Representatives of predicted interaction networks among lncRNAs and protein-coding RNAs. The triangular and foursquare nodes represent mRNAs and lncRNAs, respectively (**b**)

LncRNAs are proved to be proximal to their target genes preferentially [21–23]. In order to explore the potential functions of lncRNAs, we defined co-expressed protein-coding genes that located within 100 kb from each corresponding regulated lncRNAs as predictable targets. Differentially expressed lncRNAs were identified between two peanut RILs at the 15 DAF and 35 DAF stages, respectively, in order to dissect the lncRNAs at the seed development stage (Additional file 5: Table S5). Gene ontology (GO) analysis was performed to categorize these protein-coding genes. There were 25 classes of biological processes and these protein-coding genes were mainly enriched in “regulation of transcription”, “translation”, and “transport”. Moreover, some important growth and development-related genes were identified as lncRNAs targets, including “cell division”, “carbohydrate metabolic process”, “cell cycle”, and so on (Fig. 6a). These findings suggested that these differentially expressed lncRNAs might be involved in seed development of peanut by regulating expression of related-protein-coding genes.

To further understand the function of differentially expressed lncRNAs in seed development, putative interactive networks were constructed to disclose the relationship between lncRNAs and protein-coding genes (Fig. 6b). Among these, five protein-coding genes involved in material transportation, including protein transportation (XM_016309191.1), iron transportation (XM_016101863.1), sugar transportation (XM_016101862.1), auxin transportation (XM_016102040.1), and translation process (XM_016102046.1), were found to be regulated by sixteen lncRNAs in the seed development (Fig. 6B-1 and 6B-2). Three transcription factors, B3 domain-containing transcription factor (XM_016091512.1), squamosa promoter-binding-like protein (XM_016091385.1), and zinc-finger homeodomain protein (XM_016335443.1), were embraced in the network of Fig. 6B-3, which involved in activating growth-related genes in the downstream during seed development. In addition, several important biological processes, such as GO:0009734, auxin mediated signaling pathway; GO:0009740, gibberellic acid mediated signaling pathway; GO:0051301, cell division, also were found in this study (Additional file 5: Table S5). These results implied that the differentially expressed lncRNAs might regulate genes involved in various biological processes, including transcription, transport, hormone signal transduction, and translation controlling seed development in peanut.

**Fig. 7:**
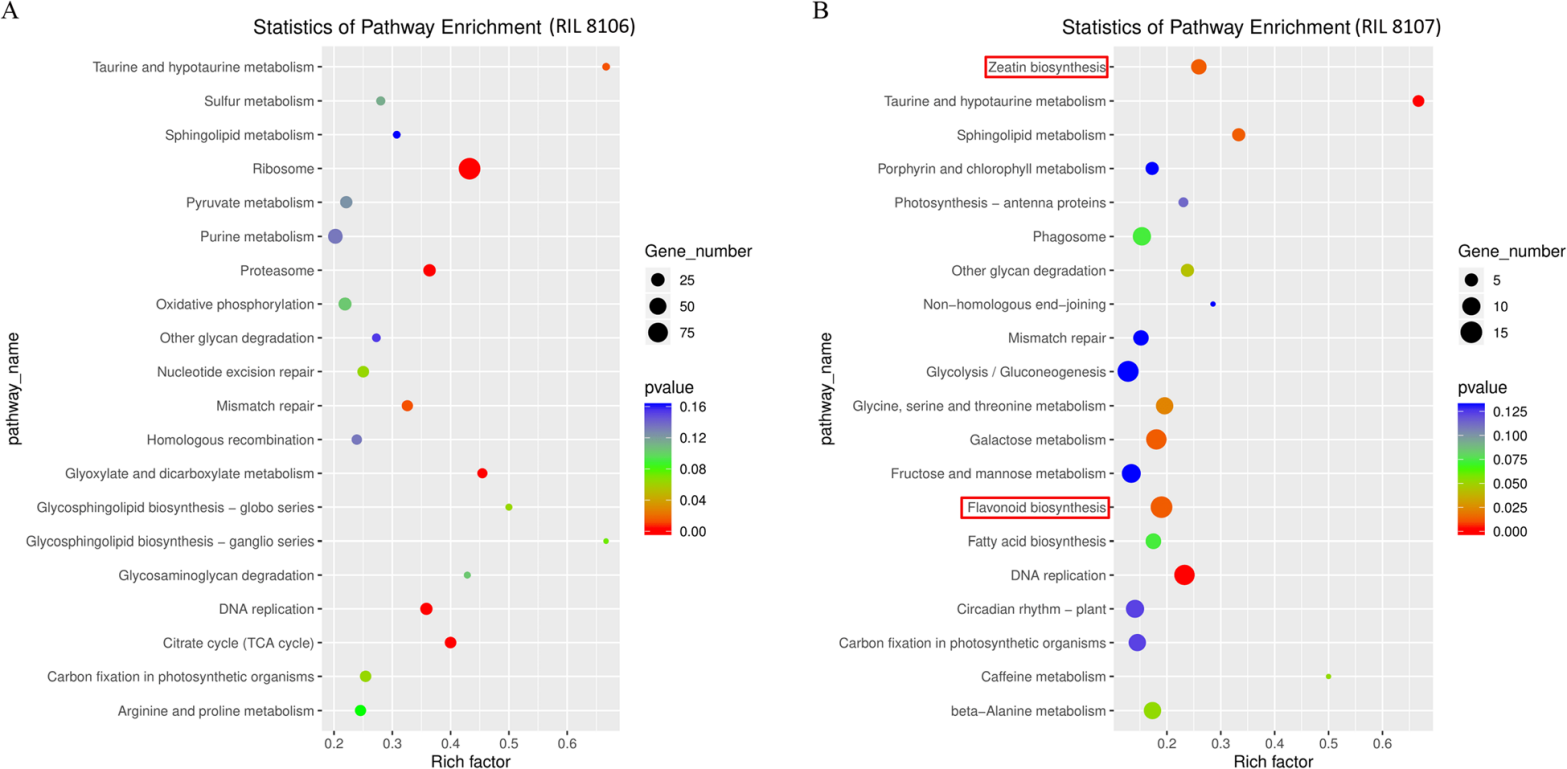
KEGG analysis of co-expressed protein-coding genes with the differentially expressed lncRNAs in RIL 8106 (**a**) and RIL 8107 (**b**)

Moreover, based on the KEGG analysis, these protein-coding genes were significantly enriched in 20 pathways in the two peanut RILs, respectively (Fig. 7a and b). Among these pathways, there are three most over-represented categories, including ribosome, purine metabolism, and oxidative phosphorylation, were significantly enriched in the medium size seed line RIL 8106. However, the three most frequent pathways are glycolysis/gluconeogenesis, flavonoid biosynthesis, and galactose metabolism in the large size seed line RIL 8107. Interestingly, some of lncRNAs target genes were found to be involved in the zeatin synthesis only in RIL8107 (Fig. 7b). In this pathway, MSTRG.13500.1, MSTRG.13501.1, and MSTRG.13501.2 were involved in zeatin biosynthesis, and the accumulation level of the CYP735A protein (Additional file 5: Table S5). These findings suggested that lncRNAs have a significant effect on the regulation of peanut seed development by effecting endogenous hormone accumulation level.

### Validation of lncRNA target genes by qPCR

**Fig. 8:**
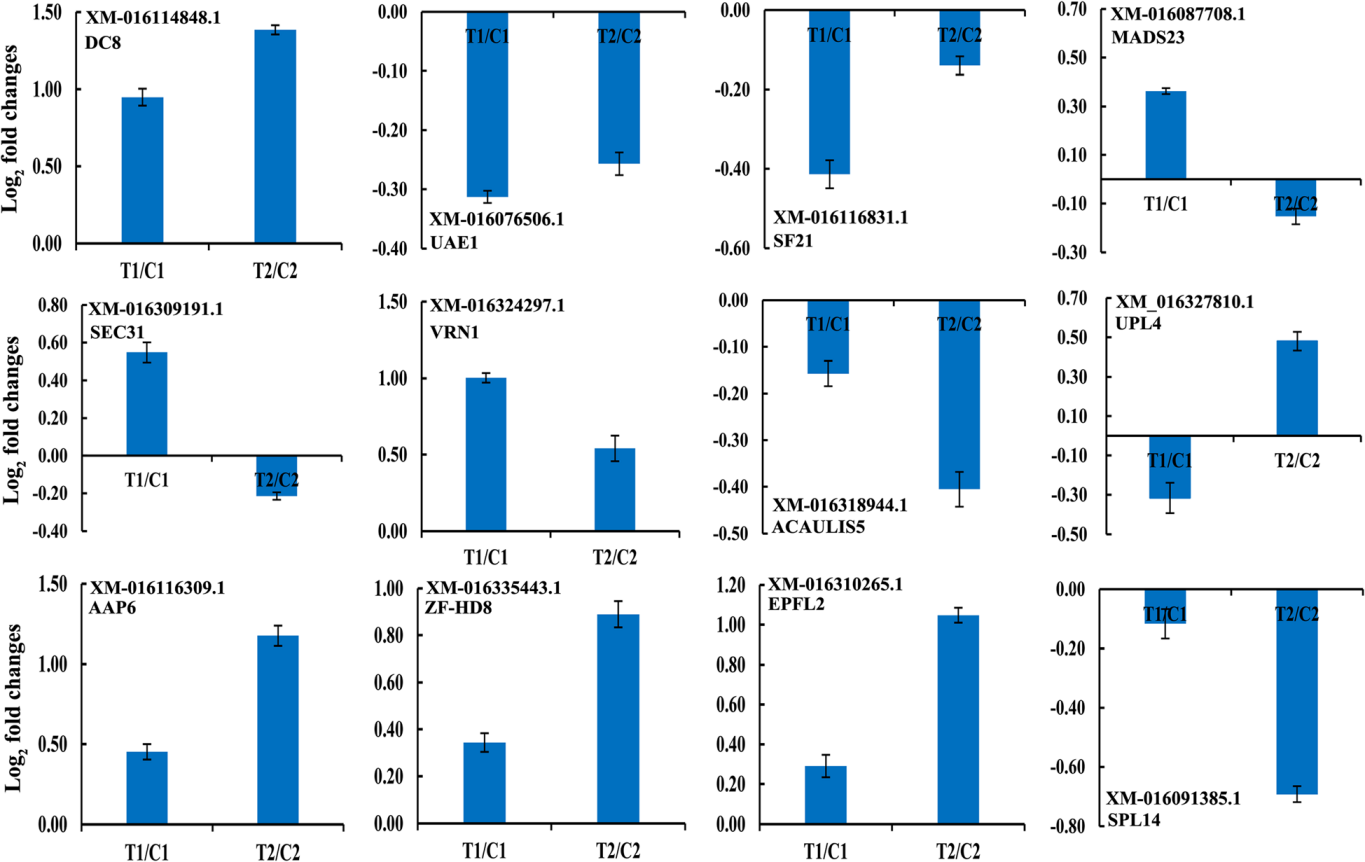
Comparison of the relative expression levels of 12 co-expressed protein-coding genes with the differentially expressed lncRNAs in two peanut RILs. The expression levels were normalized to the expression of Actin in qPCR. The log2 fold-changes in protein-coding gene expression as determined by qPCR are shown in blue. The experiments were repeated three times and vertical bars indicate the standard errors

To confirm the relationship of lncRNAs and their related protein-coding genes, the expression patterns of 12 putative cis-acting proteins as targets for 12 differentially expressed lncRNAs were verified by qPCR. Most lncRNAs and their putative cis-acting targets were co-expressed and up- or down-regulated at the 35 DAF stage (Fig. 5 and Fig. 8). The expression of lncRNA XR_001593099.1 and the target for embryonic protein DC-8-like (XM_016114848.1) were up- regulated in both peanut RILs. However, the expression of lncRNA MSTRG.18462.1 and the target for MADS-box transcription factor 23-like (XM_016087708.1), MSTRG.34915.1 and the target for protein transport protein sec31-like (XM_016309191.1), MSTRG.41848.1 and the target for B3 domain-containing transcription factor VRN1-like (XM_016324297.1) were up-regulated at the 35 DAF only in the RIL 8106. Meanwhile, the expressions of MSTRG.22884.1 and E3 ubiquitin-protein ligase UPL4 (XM_016327810.1), MSTRG.12404.1 and amino acid permease 6-like (XM_016116309.1), MSTRG.26719.1 and zinc-finger homeodomain protein 8-like (XM_016335443.1), MSTRG.35761.1 and EPIDERMAL PATTERNING FACTOR-like protein 2 (XM_016310265.1) were up-regulated at the 35 DAF only in the RIL 8107. Moreover, lncRNA MSTRG.20033.1 and squamosa promoter-binding-like protein 14 (XM_016091385.1) was down-regulated in the RIL 8107. These results indicate that lncRNAs are involved in regulating peanut seed development by modulating the expression of their cis-acting target genes.

## Discussion

### Identification of lncRNAs and verification of their functions during peanut seed development

The regulatory roles of lncRNAs are increasingly being unraveled in plants. lncRNAs have been found to be involved in plant growth, development, reproduction and abiotic stress responses in plants such as maize, rice, and Arabidopsis [24–27]. In peanut, previous studies on noncoding RNA have predominantly focused on miRNA identification and functional analysis [17, 28, 29], however, the role of lncRNAs, especially in association with peanut seed development has not been reported yet. Peanut seed development is a complex biological process regulated by coordinated gene expression. According to previous description about the whole stages of seed development in peanut [17], the first sign of pod development is seen at 15 DAF, and the pods enlarge to the maximum size at about 35 DAF, which is named the stereotyped fruit. Hence, we selected seeds at these two stages from two peanut RILs to perform a genome-wide analysis of lncRNAs using a high-throughput sequencing technology. We identified a total of 13,425 unique lncRNAs in different development stages between two peanut RILs. Of these lncRNAs, 4037 novel lncRNAs, including MSTRG.37421.1, MSTRG.18462.1, MSTRG.31185.2, MSTRG.34915.1, MSTRG.41848.1, MSTRG.22884.1, MSTRG.12404.1, MSTRG.13652.1, MSTRG.13500.1, and MSTRG.13501.1, etc., were first reported in peanut seeds, indicating their roles in seed development. Moreover, we identified 1437 differentially expressed lncRNAs at the 35 DAF stage compared to 15 DAF between two RILs. To better understand the function of differentially expressed lncRNAs in peanut seed development, we analyzed protein-coding genes that were co-expressed with these lncRNAs (Additional file 5: Table S5). Some lncRNAs co-expressed with some protein-coding genes appeared at 35 DAF stage between the two RILs regardless of seed size. For example, lncRNA XR_001593099.1 was up-regulated in both peanut RILs and the expression of the target gene of lncRNA XR_001593099.1 was also increased. The target gene, embryonic protein DC-8-like (*DC8*), has been shown to be strongly expressed during embryogenesis and in cell walls of endosperm tissues in plant [30, 31], indicating that *DC8* plays a role in peanut seed development. Our work provides an important resource of peanut lncRNAs that can be useful to the research community.

### Specific lncRNAs involved in seed development in the medium size seed line RIL 8106

In this study, many lncRNAs and their putative cis-acting target genes were co-expressed predominantly only in the RIL8106 at the 35 DAF stage. For example, three lncRNAs, MSTRG.18462.1, MSTRG.34915.1, and MSTRG.41848.1, were up-regulated only in the RIL 8106 at 35 DAF (Fig. 5). The target gene of lncRNA MSTRG.18462.1 encodes the MADS-box transcription factor 23-like (*MADS*). Previous study showed that MADS family members are key elements of the genetic networks that control flowering and fruit development [32]. TAGL1 is a transcription factor belonging to the family of the MADS-box and it has been shown to be involved in many biological processed of the fruit ripening in tomato [33]. In this work, the level of expression of the *MADS* gene was increased as the lncRNA MSTRG.18462.1 performed, which may regulate the expression of *MADS* for the process of seed development. Another up-regulated lncRNA MSTRG.34915.1 was expressed upstream of the coding region of the transport protein sec31-like (*SEC31*) at the 35 DAF stage. In Arabidopsis, *SEC* gene is proved to be essential for gametophyte development, and is required for secretory trafficking in developing pollen [34]. These results suggested that lncRNA MSTRG.34915.1 may regulate the expression of *SEC31*, which could then contribute to seed development in peanut. Similar to MSTRG.34915.1, the lncRNA MSTRG.41848.1 was located approximately 10 kb upstream of the coding region of B3 domain-containing transcription factor VRN1-like (*VRN1*) (Additional file 5: Table S5). Both of them were up-regulated at 35 DAF in RIL8106. Previous studies have demonstrated that B3 domain-containing proteins are involved in seed development, hormone response, and flowering time [35, 36]. Therefore, the lncRNA MSTRG.41848.1 could possibly be involved in regulating seed development in peanut by modulating the expression of *VRN1*. The high expression of these lncRNAs and their putative cis-acting target genes was an assumed mechanism for a specific seed development in RIL 8106.

### Specific lncRNAs involved in seed development in the large size seed line RIL 8107

A number of lncRNAs and their putative protein-coding genes was up-regulated only in RIL8107, including lncRNAs MSTRG.22884.1, MSTRG.12404.1, MSTRG.26719.1, and MSTRG.35761.1 (Fig. 5 and Fig. 8). Transcription of lncRNA MSTRG.22884.1 was significantly increased in RIL 8107 as well as its target E3 ubiquitin-protein ligase (*UPL4*). E3 family members play an important role in regulating gametogenesis and cell cycle processes during seed development in Arabidopsis [37–39]. The increased expression of the *UPL4* gene could be related with its role in peanut seed development. Another up-regulated lncRNA MSTRG.12404.1 targets amino acid permease 6-like (*AAP6*) gene, which encodes plant-specific amino acid transmembrane transporter and involved in the amino acid uptake [40]. In the present study, the expression of *AAP6* gene was up-regulated at 35 DAF, indicating that *AAP6* may also be involved in peanut seed development. The target gene of lncRNA MSTRG.26719.1 is the zinc-finger homeodomain protein 8-like (*ZF-HD8*). Previous studies have shown that *ZF-HD* genes encode a group of transcriptional regulators in floral and leaf development [41, 42]. We identified the lncRNA MSTRG.26719.1 located upstream of the coding sequence of *ZF-HD8* (Additional file 5: Table S5). The results suggested that MSTRG.26719.1 may be involved in regulating seed development by regulating the expression of *ZF-HD8*. Similarly, the target gene of another up-regulated lncRNA MSTRG.35761.1 is EPIDERMAL PATTERNING FACTOR-like protein 2 (*EPFL2*), which encode plant-specific transcription factors involved in cell differentiation. In rice, *RAE2* (an *EPFL* gene) can promote the proliferation of vasculature cells for awn elongation [43]. A similar phenomenon has been reported in Arabidopsis that *AtEPFL4* and *AtEPFL6* coordinate development of inflorescence architecture [44]. In our present work, we found that the level of *EPFL2*-specific mRNA was increased at 35 DAF stage (Fig. 5 and Fig. 8). The increased expression of *EPFL2* may also play a role in peanut seed development by regulating cell differentiation.

In contrast, the lncRNA MSTRG.20033.1 as well as its predictable target gene, squamosa promoter-binding-like protein 14 (*SPL*), were significantly down-regulated and predominantly occurred only in the RIL8107 (Fig. 5 and Fig. 8). The *SPL* plays important roles in plant developmental phase transition, flower and fruit development, gibberellins signaling etc. [45–47]. In our present study, the *SPL* gene was predicted to be regulated by the lncRNA MSTRG.20033.1, and also participated in peanut seed development simply prolonging the developmental phase change. Our findings suggest that specific regulation of these lncRNAs and their putative protein-coding genes only in RIL 8107 might explain the difference of seed size between RIL 8107 and RIL 8106.

### lncRNA-dependent regulatory networks might involve in seed development of peanut

**Fig. 9:**
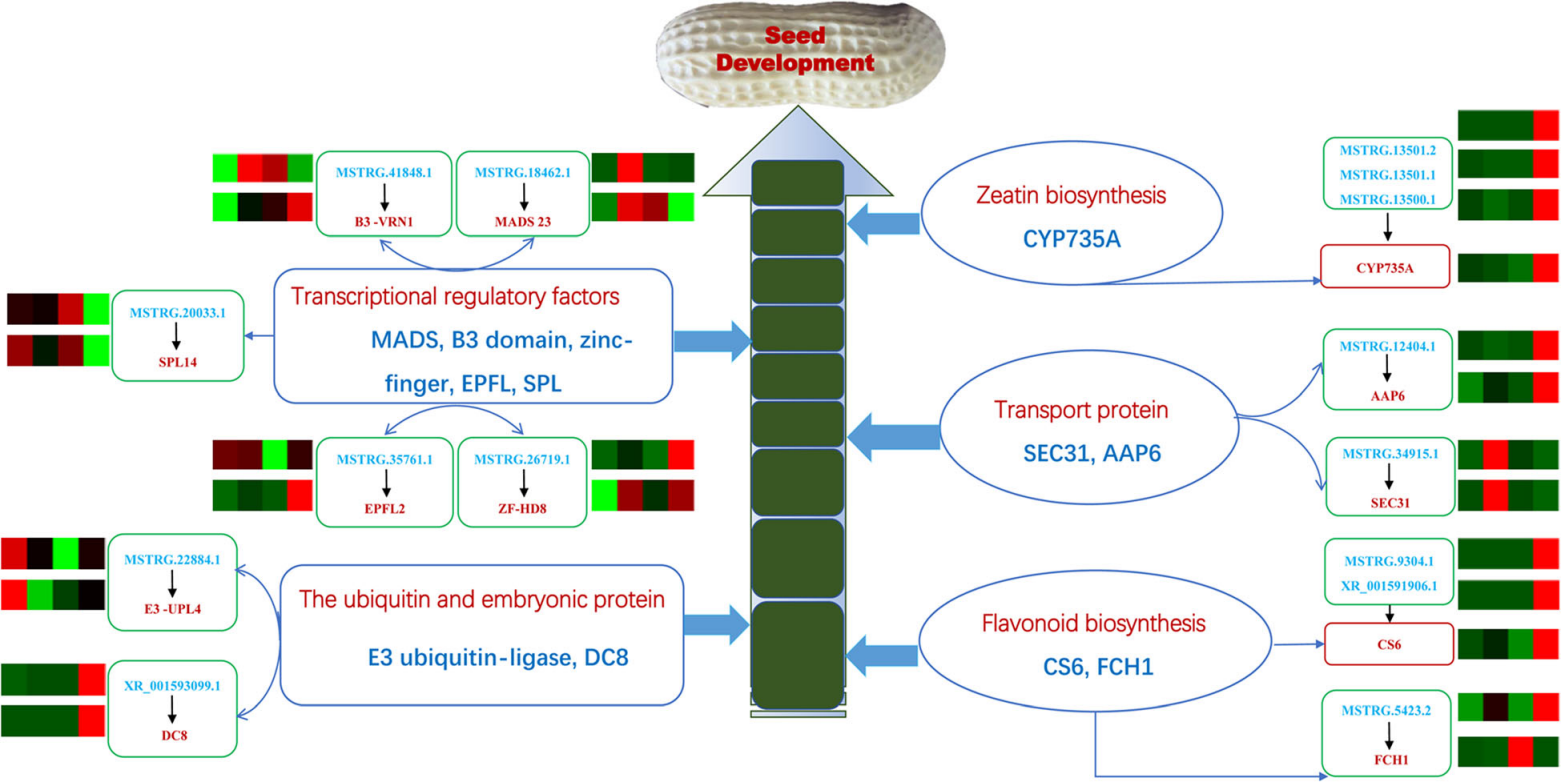
A proposed regulatory mechanism involving differentially expressed lncRNAs and their co-expressed protein-coding genes during peanut seed development. The heat map above represents the lncRNA expression pattern while the below heat map represents the mRNA of the protein-coding genes. From left to right, different colors represent C1, T1, C2, and T2

Plant endogenous hormones play vital roles in various developmental processes. Auxin regulates a vast array of growth and developmental processes in plants [48]. Cytokinins are considered to be the main hormone participating in many biological processes of the plant, such as apical dominance [49], root proliferation [50], and reproductive development [51]. Previous study has shown that *CYP735A* gene plays an important role in trans-zeatin (an endogenous cytokinin) biosynthesis in *Jatropha curcas* [52]. The Jccyp735a mutants using the CRISPR-Cas9 system has been found to have a significant decrease of the concentrations of trans-zeatin and trans-zeatin-riboside displaying severe retarded growth. In this study, we found that several lncRNAs, including MSTRG.13500.1, MSTRG.13501.1, and MSTRG.13501.2, participate in zeatin biosynthesis by regulating the expression of *CYP735A* only in RIL8107 (Fig. 7b, Fig. 9, and Additional file 5: Table S5). These findings suggest that these lncRNAs may play key roles in peanut seed development by affecting endogenous cytokinin biosynthesis. In addition, flavonoids play an important role in plant development. In Arabidopsis, the accumulation of flavonoids including flavonols, anthocyanins and proanthocyanidins in specific tissue leads to the regulation of biological processes including light-attenuation, oxidative stress protection and more importantly seed coat development [53]. In our present work, we found that lncRNAs MSTRG.9304.1 and XR_001591906.1 participate in flavonoid biosynthesis by regulating the expression of chalcone synthase 6 like (*CS6*) only in RIL8107 (Fig. 7b, Fig. 9, and Additional file 5: Table S5), indicating their potential roles in peanut seed development.

The functions of their putative protein-coding genes in peanut seed development were assessed in addition to the aforementioned lncRNAs (Fig. 9). For example, lncRNAs MSTRG.18462.1 and target *MADS23*; MSTRG.41848.1 and target *B3-VRN1*; MSTRG.22884.1 and target *E3-UPL4*; MSTRG.26719.1 and target *ZF-HD8*; MSTRG.35761.1 and target *EPFL2*; MSTRG.20033.1 and target *SPL14* were also identified to be involved in seed development. The identification of these growth and development-related lncRNAs, and the characterization of their regulatory networks can enhance the understanding of the molecular mechanisms involved in peanut and plant seed development in general.

## Conclusions

Peanut seed development is a complex process that involves a series of physiological, morphological, and transcriptional changes. This study represents the first report characterizing the expression landscape of lncRNAs involved in peanut seed development process. A total of 9388 known and 4037 novel lncRNAs were discovered. Among them, 1437 lncRNAs were differentially expressed during seed development between the two RILs. Functional analysis of the lncRNAs and their close related protein-coding genes revealed at least 11 regulatory modules of these lncRNA-mRNAs playing important roles in peanut seed development. Our results provide new insights into lncRNAs and their possible functions in peanut, as well as their expression pattern in the context of seed development; therefore, may provide new approaches for the genetic improvement of yield-related traits in peanut.

## Methods

### Plant materials

We obtained two peanut cultivars, Huayou 7 and Huayou 4, and manually crossed them to generate two eighth-generation recombinant inbred lines (RILs), RIL8106 and RIL8107. These two RILs are both erect Virginia-types with 8–10 branches, but their seed sizes displayed a main difference. RIL 8106 has smaller seeds compared with RIL8107, which was supported by a corresponding 100-seed weight of 100 g of RIL8106 and a corresponding 100-seed weight of 182 g of RIL8107. Fresh seeds were harvested from the two RILs at 15 DAF and 35 DAF, respectively, and these plants were designated as C1, C2, T1 and T2. Four seed samples were randomly chosen and subjected to liquid nitrogen before storing them at − 80 °C. For each treatment, three replicates of samples were collected.

### Construction of cDNA libraries and deep sequencing

Total RNA was extracted from each peanut seed sample using Trizol reagent (Invitrogen, CA, USA) following the manufacturer’s procedure. According to the protocol provided in the Epicentre Ribo-Zero Gold Kit (Illumina, San Diego, USA), ribosomal RNA was depleted from approximately 10 μg of total RNA representing a specific adipose type. Following purification, the poly(A)- or poly(A) + RNA fractions were cleaved into small fragments and reverse-transcribed to construct the final cDNA library based on the protocol described in the RNA-Seq sample preparation kit (Illumina, San Diego, USA). We then performed the paired-end sequencing (150 bp) on an Illumina Hiseq4000 sequencer at the LC Biotech (Hangzhou, China) following the vendor’s recommended protocol.

### Reads mapping and transcriptome assembly

Firstly, the low quality reads (including adaptor contamination, low quality bases, and undetermined bases) were removed using Cutadapt [54]. Then sequence quality was verified using FastQC (http://www.bioinformatics.babraham.ac.uk/projects/fastqc/). We then used TopHat [55] to obtain clean paired-end reads by mapping them to reference genomes of two diploid peanut species, *A. duranensis* and *A. ipaensis*, acquired from the peanutbase database (https://www.peanutbase.org/). To construct transcriptome, the mapped reads were assembled using StringTie [56]. After the final transcriptome was generated, StringTie and Ballgown [57] were used to estimate the expression levels of all transcripts.

### LncRNA identification

According to the characteristics of lncRNA, we adopted the following steps to identify lncRNAs from the transcripts of transcriptome assemblies [58]. First, all the transcripts that overlapped with reference genome were defined as “known lncRNA”. Second, among the remaining transcripts, those with length longer than 200 bp were selected for the protein-coding-score test to calculating the Coding Potential Calculator (CPC) [18] and Coding-Non-Coding Index (CNCI) [19]. Finally, the transcripts with CPC score < − 1 and CNCI score < 0 were defined as novel lncRNAs.

### Analysis of differentially expressed patterns

StringTie [56] was used to perform expression level of all transcripts, including mRNAs and putative lncRNAs by calculating FPKM [59]. Differentially expression analysis was performed using R package – Ballgown [57] with |log2 (fold change)| > 1 and *p* value < 0.05.

### Target gene functional analysis of lncRNAs

To explore the function of lncRNAs in peanut seed development, we predicted the cis-target genes of lncRNAs. The lncRNAs may play a cis role acting on neighboring target genes [60]. In this study, coding genes in 100,000 bp up- and downstream from the lncRNA, were selected by python script [61]. Moreover, we showed functional analysis of the target genes for lncRNAs by using the BLAST2GO [62] and significance was expressed as a p value < 0.05.

### Quantitative real-time PCR

We determined the transcript levels of selected lncRNAs and protein-coding genes via RT-qPCR (Additional file 6: Table S6) according to the manufacturer’s instructions for the CFX96 Real-Time System (Bio-Rad, Hercules, CA, USA) and the TB Green Premix Ex Taq II (TaKaRa, Dalian, China). Total RNA were extracted from seeds at different developmental stages using RNAsimple Total RNA Kit (TIANGEN biotech, Beijing, China), and reverse-transcribed using the PrimeScript RT reagent Kit (TaKaRa, Dalian, China). The reverse transcription reactions were performed according to the supplier’s protocol. The peanut gene Actin 7 was used as the internal control for RT-qPCR. The reactions were conducted at following conditions: 94 °C for 30 s, followed by 40 cycles of 94 °C for 5 s and 60 °C for 30 s. All reactions were performed with three replicates. The relative gene expression values were calculated by the 2^−ΔΔCT^ method [63].

## Supplementary information

**Additional file 1: Table S1**. Mean sample quality.

**Additional file 2: Table S2**. Statistical data of the RNA-Seq reads for four samples.

**Additional file 3: Table S3**. All lncRNAs in peanut.

**Additional file 4: Table S4**. Differentially expressed known and novel lncRNAs four comparisons. (S4–1) Differentially expressed lncRNAs in RIL8106 at 35 DAF (T1 vs C1). (S4–2) Differentially expressed lncRNAs in RIL8107 at 35 DAF (T2 vs C2). (S4–3) Differentially expressed lncRNAs in C2 vs C1. (S4–4) Differentially expressed lncRNAs in T2 vs T1.

**Additional file 5: Table S5**. Target genes prediction of differentially expressed lncRNAs. (S5–1) Target genes prediction of differentially expressed lncRNAs in T1 vs C1. (S5–2) Target genes prediction of differentially expressed lncRNAs in T2 vs C2.

**Additional file 6: Table S6**. All primers in this study.

## Data Availability

All data pertaining to the present study has been included in the Figures/ supplementary files of the manuscript. The two peanut recombinant inbred lines analyzed in this article (RIL 8106 and RIL 8107), are saved at Henan Agricultural University (Zhengzhou, China), and are available from the corresponding author on reasonable request. The RNA-seq datasets are available in the Sequence Read Archive (SRA) database (Accession ID: PRJNA627334; https://www.ncbi.nlm.nih.gov/sra/?term=PRJNA627334).

## Abbreviations

lncRNAs: Long noncoding RNAs
RIL: Recombinant inbred lines
DAF: Days after flowering
DELs: Differentially expressed lncRNAs
FPKM: Fragments per kilobase of exon model per million fragments mapped reads
GO: Gene ontology
KEGG: Kyoto encyclopedia of genes and genomes
CPC: Coding potential calculator
CNCI: Coding-non-coding index
